# Sex difference in the association between creatinine-to-cystatin C ratio and metabolic syndrome among Chinese adults

**DOI:** 10.3389/fendo.2024.1389295

**Published:** 2024-08-14

**Authors:** Jo-Hsuan Chen, Jau-Yuan Chen, Yi-Chuan Chen, Wen-Cheng Li

**Affiliations:** ^1^ Department of Family Medicine, Chang-Gung Memorial Hospital at Linkou, Taoyuan, Taiwan; ^2^ College of Medicine, Chang Gung University, Taoyuan, Taiwan; ^3^ Department of Health Management, Xiamen Chang-Gung Hospital, Xiamen, China

**Keywords:** serum creatinine-to-cystatin C ratio, metabolic syndrome, gender difference, adipose tissue, cystatin C

## Abstract

**Background:**

Metabolic syndrome (MetS), characterized by central obesity, insulin resistance, dyslipidemia, and hypertension, affects 20-25% of the global population. The creatinine-to-cystatin C ratio (CCR) is an indicator of skeletal muscle mass. While CCR may play a role in MetS development, sex differences in these associations are not fully understood. Therefore, this study aimed to investigate how CCR levels are associated with MetS in a Chinese adult population, focusing on possible sex disparities.

**Method:**

We conducted a retrospective cross-sectional analysis of 9,376 adults from Xiamen Chang Gung Hospital between 2014 to 2016. We examined the relationship between CCR and MetS, adjusting for cardiometabolic risk factors.

**Results:**

The prevalence of MetS was 24.7% in males and 18.0% in females. Interestingly, we observed significant sex differences in the association between CCR quartiles and MetS. Females in the lowest CCR quartile had a significantly higher risk of MetS (odds ratio=1.84). Receiver operating characteristic curve analysis revealed acceptable diagnostic power of CCR for MetS in females (area under the curve=0.65) but not in males.

**Conclusion:**

Our findings suggest that CCR is an independent risk factor for MetS in females, highlighting the importance of sex-specific assessments when evaluating MetS risk.

## 1 Introduction

Metabolic syndrome (MetS) is a cluster of integrated metabolic abnormalities, including central obesity, insulin resistance, dyslipidemia, and hypertension (1). These factors not only increase the risk of developing cardiovascular disease and type 2 diabetes (T2DM) (2), but also directly influence their incidence (3). In addition, MetS is associated with an increased risk for various other diseases, such as hyperuricemia, non-alcoholic fatty liver disease, sleep apnea, polycystic ovarian syndrome, colorectal cancer, liver cancer, pancreatic cancer, and breast cancer (3, 4).

Global statistics show a consistent rise in MetS prevalence over the past three decades, mirroring the increasing rates of T2DM and obesity worldwide. Estimates suggest that by 2018, approximately 20-25% of the global population will be affected by MetS (3, 5). In the Asia-Pacific region alone, the estimated prevalence in 2017 ranged from 11.9% to 37.1% (6). Mainland China reported an average prevalence rate of 24.2% (24.6% in men and 23.8% in women) between 2010 and 2012 (7). These figures highlight the significant public health burden of MetS, affecting both developed and developing nations.

Cystatin C (CysC) a 13 kDa protein, is considered a more ideal endogenous marker of estimated glomerular filtration rate compared to creatinine or creatinine clearance. This is because CysC is freely filtered by the glomeruli and entirely reabsorbed and catabolized in the proximal tubules, whereas creatinine undergoes partial tubular secretion (8).

Beyond its role in kidney function, CysC belongs to the cysteine protease inhibitor family and plays a part in regulating cardiovascular plaque stability (9). Research suggests a connection between CysC and the extracellular matrix of arterial walls and adipocytes (10, 11). Elevated CysC levels have been detected in various organs throughout the human body, including the lungs, brain, and adipose tissue (12).

CysC has been linked to several chronic diseases, including cardiovascular disease (13–15), early-stage renal dysfunction (8, 14), and metabolic diseases such as obesity, aging, hypertension, hyperlipidemia, diabetes (16), and MetS (17, 18). The mechanism by which CysC affects MetS is thought to be associated with renal insufficiency, insulin resistance, inflammatory and agglutination mediators, metabolic hyperactivity, and oxidative stress (11, 18). While most studies have found serum CysC concentrations to be independent of sex (19–21), some research suggests potential sex differences (22).

The Scr-to-CysC ratio (Scr/CysC ×100, CCR), also known as the sarcopenia index (23), has emerged as a biomarker for skeletal muscle mass assessment since 2013 (24). Unlike traditional methods like computed tomography or dual-energy X-ray absorptiometry, CCR offers advantages in terms of accessibility and affordability (24). Studies consistently show a correlation between CCR and muscle mass, making it a potential predictor of sarcopenia, especially in individuals with T2DM (25).

Furthermore, CCR’s denominator, CyC, is a marker of serious disease that activates cathepsin production. Therefore, a low CCR not only indicates low muscle mass but also suggests potential underlying health issues (26, 27). The clinical utility of CCR appears promising and warrants further investigation across various pathological conditions (23). However, guidelines or consensus regarding its use have not been definitively established.

Previous research suggests that estrogen and testosterone influence muscle synthesis and fat distribution (28). However, the impact of sex on the association between CCR and MetS remains inconclusive. Therefore, this study aims to examine the correlation between CCR and MetS, with a specific focus on potential sex differences in this relationship.

## 2 Materials and methods

### 2.1 Study designs and participants

This retrospective cross-sectional study was conducted at Xiamen Chang Gung Hospital from January 2014 to December 2016. We retrospectively collected data from all participants aged >18 years old who underwent standard clinical evaluation and blood biochemistry testing at the hospital’s Physical Examination Center. Self-reported health questionnaires were completed at the center. Physical examination and venous blood sampling were then conducted if participants had fasted for more than 12 hours or self-reported not being pregnant. Trained research nurses assisted participants throughout the process.

Exclusion criteria were applied to eliminate factors that could affect metabolic test results or body composition. These included: (1) current use of steroids, which can affect metabolism (n=45); and (2) chronic diseases that could significantly impact CCR levels or metabolism, such as cardiovascular disease (including heart failure, myocardial infarction, and stroke [n=18]), chronic hepatitis and cirrhosis (n=86), and thyroid disease (n=49). The study protocol was approved by the Xiamen Chang Gung Medical Foundation Institutional Review Board (IRB numbers: XMCGIRB2022103). All methods adhered to relevant guidelines and regulations. Ultimately, 9,376 participants, comprising 5,222 men and 4,154 women, were deemed eligible for analysis.

### 2.2 Data collection

We systematically gathered data on various covariates, including demographics (age and sex), anthropometry (height, weight, and waist circumference), blood pressure (BP), total cholesterol (TC), low-density lipoprotein cholesterol (LDL-C), high-density lipoprotein cholesterol (HDL-C), triglyceride (TG), fasting blood glucose, serum creatine (Scr), and serum CysC. Participants self-reported their medical history, including current diseases and medications, which were documented in a standardized format.

### 2.3 MetS definition

We diagnosed MetS according to the Third Adult Treatment Panel criteria of the National Cholesterol Education Program. A participant was classified as having MetS if they met at least three of the following five criteria: 1) high BP (systolic BP [SBP] ≥130 mmHg and/or diastolic BP [DBP] ≥85 mmHg, under treatment, or previously diagnosed with hypertension); 2) high serum TGs (≥1.7mmol/L or under treatment); 3) decreased HDL-C (<1.03mmol/L for males and <1.29mmol/L for females, or under treatment); 4) hyperglycemia (fasting blood glucose ≥5.6mmol/L, under treatment, or previously diagnosed with T2DM); and 5) abdominal obesity (waist circumference cutoffs were modified for Asian populations, ≥90 cm for men and ≥80 cm for women). A participant was diagnosed with MetS if they had a waist circumference exceeding the threshold along with two other risk factors, or a waist circumstance within the threshold but with three or more other risk factors.

### 2.4 Assessment of potential covariates

Information on covariates like age, sex, pregnancy status, and comorbidities (hypertension, ischemic heart disease, acute infection, liver disease, T2DM, tumor, etc.) was collected through hospital information systems and face-to-face interviews. Body weight and height were measured following standard protocols. Waist circumference was measured at the midpoint between the iliac crest and the lowest rib. Body mass index (BMI) was calculated as weight divided by height squared (kg/m²). BP was measured three times using an automated sphygmomanometer after participants rested in a seated position for 15 minutes. The average of the readings was used for SBP and DBP. Mean arterial pressure (MAP) was calculated using the formula: (2/3) × DBP + (1/3) × SBP. We adjusted for potential confounders by performing three models of MetS analysis: Model 1, unadjusted analysis; Model 2, adjusted for age and fasting glucose level; and Model 3, adjusted for variables in Model 2, plus TG, HDL-C, and LDL-C levels.

Fasting plasma glucose was measured using a modified hexokinase enzymatic assay (Cobas Mira Chemistry System; Roche Diagnostic Systems, Montclair, New Jersey, USA). The biochemical autoanalyzer (DxC 800, Beckman Coulter UniCel DxCSYNCHRON, Ireland) was used to measure TC, HDL-C, and TG. Blood samples were collected by experienced nurses after a minimum 12-hour fast.

### 2.5 Measurement of CysC, Scr, and CCR

Scr concentration (mg/dL) and CysC concentration (mg/L) were measured using a turbidimetric immunoassay on the Abbott ARCHITECT c8000/c16000 analyzer (Abbott Laboratories, Abbott Park, Illinois, USA). Renal function was assessed by calculating CCR using the formula: Scr (mg/dL)/CysC (mg/L) × 100. Participants were then divided into four groups based on their CCR quartiles: Q1 (lowest), Q2, Q3, and Q4 (highest).

### 2.6 Statistical analysis

Parametric continuous variables were expressed as mean ± standard deviation (SD). Categorical data were presented as frequencies (percentages). Differences between groups for categorical variables were assessed using the chi-square test. Student’s t-test was used for normally distributed continuous variables, while the Mann-Whitney U test was used for non-normally distributed variables. Additionally, a one-way analysis of variance was used to assess differences between groups for continuous factors, followed by a Bonferroni *post-hoc* test for pairwise comparisons if the overall relationship was significant.

The relationship between MetS risk factors and CCR quartiles was examined using both univariate and multivariate logistic regression analyses. Results are presented as odds ratios (OR) with their corresponding 95% confidence intervals (CI). Receiver operating characteristic curves were generated to evaluate the cut-off point value and predictive power of CCR for MetS diagnosis. All statistical analyses were performed using SPSS version 25.0 (SPSS, Chicago, IL, USA). A two-tailed *p* value <0.05 was considered statistically significant.

## 3 Results

Our analysis included 5,222 men and 4,154 women with an average age of 47 years in both groups. The prevalence of MetS was 24.65% in men and 17.98% in women. **Table 1** summarizes the baseline characteristics, including cardiometabolic risk factors. Men had significantly higher mean values (*p*-value <0.001) compared to women in BMI, waist circumference, waist-to-height ratio, MAP, fasting glucose, TC, TG, LDL-C, HDL-C, and TG/HDL-C ratio. Additionally, serum creatinine, CysC, and the CCR, were all significantly higher in men (96.03 ± 17.52 µmol/mg vs. 85.76 ± 17.00 µmol/mg).

**Table 1:** Basic characteristics of the study subjects.

Variables	Total	Men	Women	*p* value
	(n=9376)	(n=5222)	(n=4154)		
Age (year)	47.56 ± 10.47	47.40 ± 10.35	47.77 ± 10.61	0.09
BMI (kg/m^2^)	23.80 ± 3.32	24.44 ± 3.22	23.00 ± 3.27	<0.001
Waist circumference (cm)	82.89 ± 9.71	86.38 ± 8.79	78.49 ± 8.99	<0.001
Waist-to-height ratio	0.51 ± 0.06	0.51 ± 0.05	0.50 ± 0.06	<0.001
Mean arterial pressure (mmHg)	87.39 ± 13.37	90.36 ± 12.79	83.66 ± 13.15	<0.001
Fasting glucose (mmol/L)	5.31 ± 1.34	5.43 ± 1.52	5.20 ± 1.13	<0.001
Total cholesterol (mmol/L)	5.23 ± 0.98	5.28 ± 1.02	5.15 ± 1.02	<0.001
Triglycerides (mmol/L)	1.55 ± 1.30	1.85 ± 1.54	1.26 ± 0.82	<0.001
LDL cholesterol (mmol/L)	3.36 ± 0.86	3.48 ± 0.91	3.22 ± 0.89	<0.001
HDL cholesterol (mmol/L)	1.32 ± 0.32	1.15 ± 0.36	1.41 ± 0.50	<0.001
TG/HDL-C	72.87 ± 13.88	1.76 ± 1.57	1.06 ± 0.89	<0.001
Scr (µmol/L)	1.36 ± 1.58	81.59 ± 11.31	61.90 ± 7.62	<0.001
CysC (mg/L)	0.92 ± 0.21	0.99 ± 0.20	0.85 ± 0.19	<0.001
CCR	91.48 ± 18.03	96.03 ± 17.52	85.76 ± 17.00	<0.001
Metabolic syndrome, n (%)	2034 (21.69%)	1287 (24.65%)	747 (17.98%)	<0.001

**Table 2** presents the correlation and trend analyses between different CCR quartiles and various parameters in both male and female groups. In females, there was a significant difference (*p <*0.05) and an incremental increase (*p* for trend <0.05) across CCR quartiles for age, BMI, waist-to-hip ratio, MAP, fasting glucose, TC, LDL-C, HDL-C, Scr, CysC, CCR, and MetS prevalence. For males, age, BMI, waist-to-hip ratio, waist circumference, MAP, fasting glucose, TC, TG, LDL-C, HDL-C, TG/HDL-C, Scr, CysC, CCR, and MetS all showed significant associations with CCR levels (*p <*0.05). Notably, MAP and TGs did not exhibit an incremental increase across CCR quartiles in males.

**Table 2:** Correlation of sex-specific creatinine-to-cystatin C ratio level with different cardiometabolic risk factors.

Variables	CCR quartile1	CCR quartile 2	CCR quartile 3	CCR quartile 4	*p* value	*p* trend
**Men**	(<84.23)	(84.23-95.40)	(95.41-106.85)	(>106.85)		
Number	n=1305	n=1305	n=1306	n=1306		
Age (year)	52.17 ± 11.43	47.81 ± 9.94 ^a^	45.33 ± 9.29 ^a,b^	44.28 ± 8.70 ^a,b,c^	<0.001	<0.001
BMI (kg/m^2^)	24.23 ± 3.41	24.43 ± 3.32	24.49 ± 3.14	24.62 ± 2.97 ^a^	0.02	<0.001
Waist circumference (cm)	86.73 ± 9.61	86.68 ± 8.81	86.17 ± 8.56	85.94 ± 8.10	0.05	0.01
Waist-to-height ratio	0.52 ± 0.06	0.51 ± 0.05	0.51 ± 0.05 ^a^	0.51 ± 0.05 ^a,b^	<0.001	<0.001
Mean arterial pressure (mmHg)	91.28 ± 13.83	89.75 ± 12.08 ^a^	90.24 ± 12.56	90.16 ± 12.58	0.02	0.07
Fasting glucose (mmol/L)	5.27 ± 1.02	5.38 ± 1.40	5.42 ± 1.56	5.60 ± 1.86 ^a,b,c^	<0.001	<0.001
Total cholesterol (mmol/L)	5.15 ± 0.98	5.30 ± 0.97 ^a^	5.31 ± 0.98 ^a^	5.40 ± 0.98 ^a,b^	<0.001	<0.001
Triglycerides (mmol/L)	1.86 ± 1.66	1.84 ± 1.47	1.77 ± 1.27	1.85 ± 1.66	0.40	0.63
LDL cholesterol (mmol/L)	3.38 ± 0.86	3.48 ± 0.85 ^a^	3.53 ± 0.87 ^a^	3.53 ± 0.86 ^a^	<0.001	<0.001
HDL cholesterol (mmol/L)	1.17 ± 0.29	1.21 ± 0.27 ^a^	1.22 ± 0.27	1.25 ± 0.28 ^a,b,c^	<0.001	<0.001
TG/HDL-C	1.80 ± 2.21	1.70 ± 1.70	1.62 ± 1.64	1.67 ± 1.99	0.09	0.04
Scr (µmol/L)	77.85 ± 11.37	79.83 ± 9.65 ^a^	82.55 ± 10.30 ^a,b^	86.13 ± 12.02 ^a,b,c^	<0.001	<0.001
CysC (mg/L)	1.19 ± 0.21	1.00 ± 0.12 ^a^	0.93 ± 0.12 ^a,b^	0.83 ± 0.12 ^a,b,c^	<0.001	<0.001
CCR	74.82 ± 7.96	89.86 ± 3.21 ^a^	100.89 ± 3.30 ^a,b^	118.55 ± 11.18 ^a,b,c^	<0.001	<0.001
Metabolic syndrome, n (%)	364 (27.89%)	319 (24.44%)	306 (23.43%)	298 (22.82%) ^a^	0.01	0.002
**Women**	(<74.78)	(74.78-84.94)	(84.95-95.69)	(>95.69)		
Number	n=1038	n=1038	n=1038	n=1040		
Age (year)	53.93 ± 10.84	48.02 ± 10.37 ^a^	45.81 ± 9.87 ^a,b^	43.33 ± 8.17 ^a,b,c^	<0.001	<0.001
BMI (kg/m^2^)	24.08 ± 3.70	23.03 ± 3.13 ^a^	22.63 ± 3.03 ^a,b^	22.24 ± 2.89 ^a,b,c^	<0.001	<0.001
Waist circumference (cm)	82.12 ± 9.88	78.88 ± 8.52 ^a^	77.36 ± 8.23 ^a,b^	75.62 ± 7.92 ^a,b,c^	<0.001	<0.001
Waist-to-height ratio	0.53 ± 0.07	0.50 ± 0.06 ^a^	0.49 ± 0.06 ^a,b^	0.48 ± 0.05 ^a,b,c^	<0.001	<0.001
Mean arterial pressure (mmHg)	88.28 ± 13.93	84.10 ± 13.53 ^a^	81.89 ± 12.45 ^a,b^	80.38 ± 11.18 ^a,b,c^	<0.001	<0.001
Fasting glucose (mmol/L)	5.29 ± 1.06	5.13 ± 0.83 ^a^	5.15 ± 1.04 ^a^	5.16 ± 1.40	0.01	0.02
Total cholesterol (mmol/L)	5.25 ± 0.96	5.15 ± 0.99	5.16 ± 0.98	5.06 ± 0.96 ^a^	<0.001	<0.001
Triglycerides (mmol/L)	1.37 ± 0.92	1.26 ± 0.81 ^a^	1.14 ± 0.75 ^a,b^	1.05 ± 0.74 ^a,b,c^	<0.001	<0.001
LDL cholesterol (mmol/L)	3.30 ± 0.84	3.21 ± 0.86	3.21 ± 0.84	3.12 ± 0.81 ^a^	<0.001	<0.001
HDL cholesterol (mmol/L)	1.39 ± 0.30	1.43 ± 0.29 ^a^	1.48 ± 0.31 ^a,b^	1.52 ± 0.30 ^a,b,c^	<0.001	<0.001
TG/HDL-C	1.10 ± 1.01	0.98 ± 0.87 ^a^	0.86 ± 0.75 ^a,b^	0.76 ± 0.73 ^a,b,c^	<0.001	<0.001
Scr (µmol/L)	60.15 ± 9.14	61.95 ± 6.29 ^a^	62.58 ± 8.04 ^a^	62.90 ± 6.36 ^a,b^	<0.001	<0.001
CysC (mg/L)	1.05 ± 0.20	0.87 ± 0.10 ^a^	0.79 ± 0.10 ^a,b^	0.67 ± 0.09 ^a,b,c^	<0.001	<0.001
CCR	65.42 ± 8.03	80.15 ± 2.87 ^a^	107.47 ± 11.78 ^a,b^	107.47 ± 11.78 ^a,b,c^	<0.001	<0.001
Metabolic syndrome, n (%)	311 (29.96%)	193 (18.59%) ^a^	149 (14.35%) ^a^	94 (9.04%) ^a,b,c^	<0.001	<0.001

^a^p < 0.05 vs. SI quartile 1. ^b^p < 0.05 vs. SI quartile 2. ^c^p < 0.05 vs. SI quartile 3 in the Bonferroni post-hoc comparisons.

We used three models to examine the relationship between CCR quartiles and MetS, stratified by sex. Model 1 included no adjustments, Model 2 adjusted for age and fasting glucose, and Model 3 adjusted for all variables in Model 2, plus TG, HDL-C, and LDL-C levels. Multivariate logistic regression was used to analyze the relationship between sex-specific CCR quartiles and MetS based on these models.

**Table 3** shows that in females, the lowest CCR quartile (quartile 1) was significantly related to MetS in all models (p <0.001). This association remained significant even after full adjustment in Model 3 (OR 1.84, p=0.002, 95% CI 0.82-1.32). Conversely, in males, the initial significant correlation between CCR and MetS was no longer present after adjustments were made.

**Table 3:** Logistic regression analysis of creatinine-to-cystatin C ratio level and metabolic syndrome models.

Variables	Mets, n (%)		Model 1			Model 2			Model 3		
			OR	(95% CI)	*p* value	OR	(95% CI)	*p* value	OR	(95% CI)	*p* value
Men (n=5222)											
CCR quartile 4	298	(22.82%)	Reference			Reference			Reference		
CCR quartile 3	306	(23.43%)	1.04	(0.86-1.24)	0.71	1.16	(0.95-1.41)	0.14	1.02	(0.81-1.28)	0.90
CCR quartile 2	319	(24.44%)	1.09	(0.91-1.31)	0.33	1.24	(1.02-1.51)	0.03	0.99	(0.78-1.24)	0.90
CCR quartile 1	364	(27.89%)	1.31	(1.10-1.56)	0.003	1.55	(1.27-1.89)	<0.001	1.04	(0.82-1.32)	0.73
*p* value for trend			0.002			<0.001			0.80		
Women (n=4154)											
CCR quartile 4	94	(9.04%)	Reference			Reference			Reference		
CCR quartile 3	149	(14.35%)	1.69	(1.28-2.22)	<0.001	1.58	(1.16-2.16)	<0.001	1.31	(0.88-1.93)	0.18
CCR quartile 2	193	(18.59%)	2.30	(1.77-2.99)	<0.001	2.05	(1.52-2.77)	<0.001	1.40	(0.96-2.04)	0.08
CCR quartile 1	311	(29.96%)	4.31	(3.35-5.53)	<0.001	2.67	(1.98-3.60)	<0.001	1.84	(1.26-1.68)	0.002
*p* value for trend			<0.01			<0.001			0.001		

**Figure 1:**
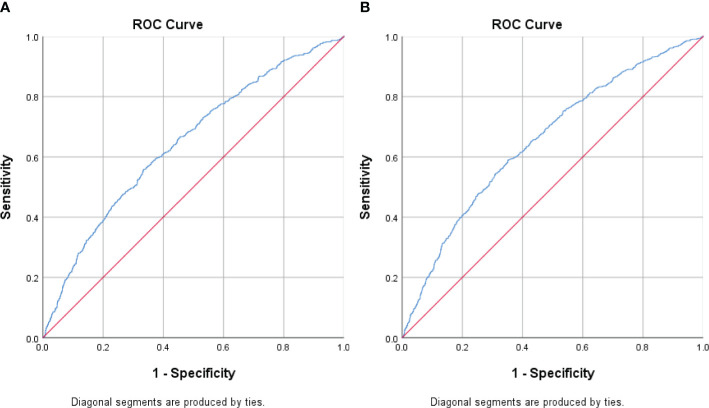
Receiver Operating Curve (ROC) analyses for creatinine-to-cystatin C ratio (CCR) levels as a predictor of metabolic syndrome stratified by sex. **(A)** Men **(B)** Women. The ROC curve indicates that CCR levels could potentially act as a marker for metabolic syndrome, especially in females.

We performed receiving operator characteristic curve analysis to determine CCR cut-off values for detecting MetS in males and females. The results are presented in **Table 4**, **Figure 1**. For males, Youden’s index identified a cut-off point of 96.72. However, the area under the curve was 0.53 (95% CI 0.52-0.54), indicating only moderate discriminatory power. Additionally, the sensitivity was 57.3% and the specificity was 47.9%. In contrast, the female group showed a CCR cut-off value of 80.95 with an area under the curve of 0.65 (95% CI 0.64-0.67), indicating acceptable discriminatory power. Furthermore, the sensitivity was 58.9% and the specificity was 64.8%. These findings suggest that CCR may be a better diagnostic tool for MetS in females compared to males.

**Table 4:** Cut-off value and prediction power for sex-specific creatinine-to-cystatin C ratio level.

Variables	AUC	(95% CI)	p value	Cut-off point according to Youden’s index	Sensitivity (%)	Specificity (%)
Male (n=5954)						
CCR level	0.53	(0.52 to 0.54)	0.001	96.72	57.3	47.9
Female (n=4185)						
CCR level	0.65	(0.64 to 0.67)	<0.001	80.95	58.9	64.8

## 4 Discussion

Our large-scale study is the first to investigate how the relationship between MetS and CCR levels varies across sexes. In females, we found a notable correlation between CCR levels and MetS, with a potential threshold of 80.95. This suggests that CCR may be a valuable biomarker for detecting and potentially mitigating MetS risk in women. These insights are crucial for healthcare providers and policymakers, informing the development of sex-specific interventions and management approaches for MetS.

The CCR ratio reflects the balance between fat and muscle mass. Patients with MetS, particularly women, may benefit from incorporating resistance training alongside aerobic exercise to build muscle mass and improve their CCR ratio. Additionally, a protein-rich diet can further contribute to muscle mass gain, potentially lowering MetS risk in females. Given the strong association between MetS and cardiovascular risk, the Pooled Cohort Risk Assessment Equation can be used alongside CCR in females for comprehensive risk assessment and cardiovascular event prediction (29).

Since CCR can serve as a sarcopenia index, women with sarcopenia may be more susceptible to MetS development compared to men. Studies suggest a stronger link between muscle mass loss and insulin resistance in women (30, 31). However, some research indicates that age and sex might not be independent risk factors for both CCR and T2DM (32–34).

For example, a study by Komorita et al. found that males had higher average CCR values compared to postmenopausal females (0.94 ± 0.14 vs. 0.73 ± 0.12). Interestingly, males with higher CCR quartiles, exhibited significant reductions in calcium intake, glycated hemoglobin levels, and insulin therapy rates. In contrast, postmenopausal females with higher CCR quartiles had a lower average BMI and a lower incidence of fragility fractures. Notably, CCR remained strongly correlated with fractures even after adjusting for BMI and blood glucose levels (35).

The higher CCR in males might be linked to hormonal differences, such as higher insulin-like growth factor-1 and testosterone levels (36). Moreover, sex disparities in fat accumulation and distribution could also play a role. Women naturally tend to store more fat, potentially as an energy reserve for future pregnancy and lactation (37). Estrogen further influences white adipose tissue distribution, promoting subcutaneous fat accumulation and inhibiting the expansion of visceral fat (38). More research is required to definitively understand the impact of sex on CCR.

Qiu et al. conducted a four-year longitudinal study in elderly Chinese individuals, finding that higher CCR was associated with a reduced risk of T2DM in a non-linear fashion. The study also observed correlations between CCR and BP, glycated hemoglobin, blood lipids, and C-reactive protein (CRP) (32). Another study by Qiu et al. involving over 5,000 healthy Chinese elderly participants, showed an inverse relationship between CCR and both BMI and waist-to-hip ratio in men. Additionally, higher CCR was linked to a significant decrease in T2DM risk. The study also highlighted a non-linear association between normalized CCR measures and T2DM (33). The potential mechanism for the association between CCR and blood glucose is thought to be related to sarcopenia, as skeletal muscle plays a crucial role in regulating blood sugar levels after meals (25, 39, 40). Reduced muscle glucose uptake can lead to abnormal carbohydrate metabolism and high blood glucose levels (25, 40).

CysC levels may also be linked to metabolic health. Its gene is highly expressed in adipose tissues, and obesity can increase CysC production by two to threefold (12). Subcutaneous and omental fat tissues significantly overexpress CysC, impacting adipose tissue and vascular health by inhibiting cathepsins, enzymes that influence fat tissue function and contribute to obesity-related issues.

Uygur et al.’s study highlighted the significance of epicardial adipose tissue (EAT) volume as a predictor of cardiovascular events in T2DM patients. They found that patients with higher EAT volumes had a significantly higher incidence of major adverse cardiac events (41).

A longitudinal study by Magnusson et al. involving 28,449 subjects over 16 years demonstrated that CysC levels can predict the onset of MetS. This correlation was significantly associated with visceral fat, suggesting that increased baseline CysC levels are linked to the development and long-term progression of abdominal obesity (34).

An et al.’s research supports the use of CCR as a tool for body composition assessment. Currently, the understanding of the relationship between CCR and adiposity is mainly based on CysC levels (42). However, further research is needed to definitively establish the role of CCR in reflecting visceral fat and muscle mass.

Increased fat tissue contributes to oxidative stress and inflammation, which can promote insulin resistance and dyslipidemia. Studies have shown a positive correlation between CysC and TG, TC, and LDL-C, along with a negative association with HDL-C. Salman et al. reported this association in children and adolescents (43), while Harada et al. found a link between high CysC levels and high TG/HDL-C ratios in Japanese boys aged 12-15 (44).

The proposed mechanism involves several steps. Fat accumulation leads to hyperinflation, increased glomerular permeability, and glomerular hypertension. These factors promote oxidative stress, inflammation, apoptosis, and renal scarring. Additionally, the reabsorption of cholesterol and fatty acids by tubular epithelial cells induces tubulointerstitial inflammation. This inflammation stimulates the formation of foam cells and damages glomerular cells. Furthermore, lipoprotein deposition in mesangial cells may promote extracellular matrix components, ultimately leading to glomerulosclerosis (45).

Chronic kidney disease creates a vicious cycle. Systemic mineral imbalance, or hyperphosphatemia, promotes atherosclerosis progression (46). A 2022 Japanese study by Y. Hashimoto found a positive correlation between CCR and subclinical atherosclerosis prevalence in individuals with T2DM (47). Vascular calcification increases arterial stiffness, contributing to systolic hypertension development (48). Furthermore, CysC levels positively correlated with SBP and DBP. Elevated BP damages the intrarenal vasculature, leading to renal ischemia and glomerulosclerosis (49). MetS components, including obesity, insulin resistance, dyslipidemia, and hypertension, individually increase the risk of renal dysfunction. In obesity, inflammatory cytokine increase and compression of the renal hilum by visceral adipose tissue activate the renin-angiotensin-aldosterone system (50). The combination of low muscle mass and high visceral fat tissue may lead to higher SBP in elderly females. This is because they have lower muscle mass and higher adipose tissue content compared to males (51).

Our study has several limitations. The cross-sectional design restricts our ability to establish a causal relationship between CCR and MetS. Prospective longitudinal cohort studies are necessary to definitively confirm this association. Additionally, our study population consisted of healthy participants undergoing routine checkups, limiting the generalizability of our findings to patient populations or other clinical settings.

Furthermore, we did not account for potential confounding factors like smoking or CRP levels, which can influence CysC concentration (52). A small study showed an inverse association between CysC and angiotensin II receptor blocker use (53). However, we could not exclude participants using ACE inhibitors due to MetS definitions. The impact of inflammation on CysC levels was also not assessed, and muscle mass was estimated using BMI instead of a more precise method like bioelectrical impedance analysis.

Further research can address these limitations. Longitudinal studies can establish causality and provide a clearer picture of the temporal relationship between sex and MetS development. Increased sample size and diversity would enhance the generalizability and statistical power of the research. Analyses based on confounders like age, BMI, comorbidities, and lifestyle factors could identify specific subgroups where sex might be a more reliable biomarker for MetS risk. This approach would prevent overlooking important subgroups due to generalized findings and could lead to more tailored treatment strategies for specific populations.

## 5 Conclusions

Our study in Chinese adults identified a correlation between serum CCR levels and MetS in females. CCR may also serve as a potential predictor of sarcopenia. Including CCR analysis in routine medical examinations, particularly for women, could be advantageous for early detection of MetS.

## Data Availability

The original contributions presented in the study are included in the article/supplementary material. Further inquiries can be directed to the corresponding author.
