# Corrigendum: Sex difference in the association between creatinine-to-cystatin C ratio and metabolic syndrome among Chinese adults

**DOI:** 10.3389/fendo.2024.1502904

**Published:** 2024-10-17

**Authors:** Jo-Hsuan Chen, Jau-Yuan Chen, Yi-Chuan Chen, Wen-Cheng Li

**Affiliations:** ^1^ Department of Family Medicine, Chang-Gung Memorial Hospital at Linkou, Taoyuan, Taiwan; ^2^ College of Medicine, Chang Gung University, Taoyuan, Taiwan; ^3^ Department of Health Management, Xiamen Chang-Gung Hospital, Xiamen, China

**Keywords:** serum creatinine-to-cystatin C ratio, metabolic syndrome, gender difference, adipose tissue, cystatin C

In the published article, there was an error. A mistake was made with the IRB number.

A correction has been made to Materials and Methods section, 2.1 Study designs and participants, second paragraph, seventh line. This sentence previously stated:

“The study protocol was approved by the Xiamen Chang Gung Medical Foundation Institutional Review Board (IRB numbers: AF-IRB-007-01.0). All methods adhered to relevant guidelines and regulations.”

The corrected sentence appears below:

“The study protocol was approved by the Xiamen Chang Gung Medical Foundation Institutional Review Board (IRB numbers: XMCGIRB2022103). All methods adhered to relevant guidelines and regulations.”

The authors apologize for this error and state that this does not change the scientific conclusions of the article in any way. The original article has been updated.

